# Personalized medicine: risk prediction, targeted therapies and mobile health technology

**DOI:** 10.1186/1741-7015-12-37

**Published:** 2014-02-28

**Authors:** Daniel F Hayes, Hugh S Markus, R David Leslie, Eric J Topol

**Affiliations:** 1 University of Michigan School of Public Health, Ann Arbor, Michigan, USA; 2 Department of Clinical Neurosciences, University of Cambridge, Cambridge, UK; 3 Department of Diabetes, St Bartholomew’s Hospital and Blizard Institute, London, UK; 4 Scripps Translational Science Institute, La Jolla, California, USA

**Keywords:** Diabetes, Genetics, Mobile health, Oncology, Personalized medicine, Smartphone, Stroke, Targeted therapy

## Abstract

Personalized medicine is increasingly being employed across many areas of clinical practice, as genes associated with specific diseases are discovered and targeted therapies are developed. Mobile apps are also beginning to be used in medicine with the aim of providing a personalized approach to disease management. In some areas of medicine, patient-tailored risk prediction and treatment are applied routinely in the clinic, whereas in other fields, more work is required to translate scientific advances into individualized treatment. In this forum article, we asked specialists in oncology, neurology, endocrinology and mobile health technology to discuss where we are in terms of personalized medicine, and address their visions for the future and the challenges that remain in their respective fields.

## Progress and challenges in individualizing cancer treatment

Daniel F Hayes

**Figure 1:**
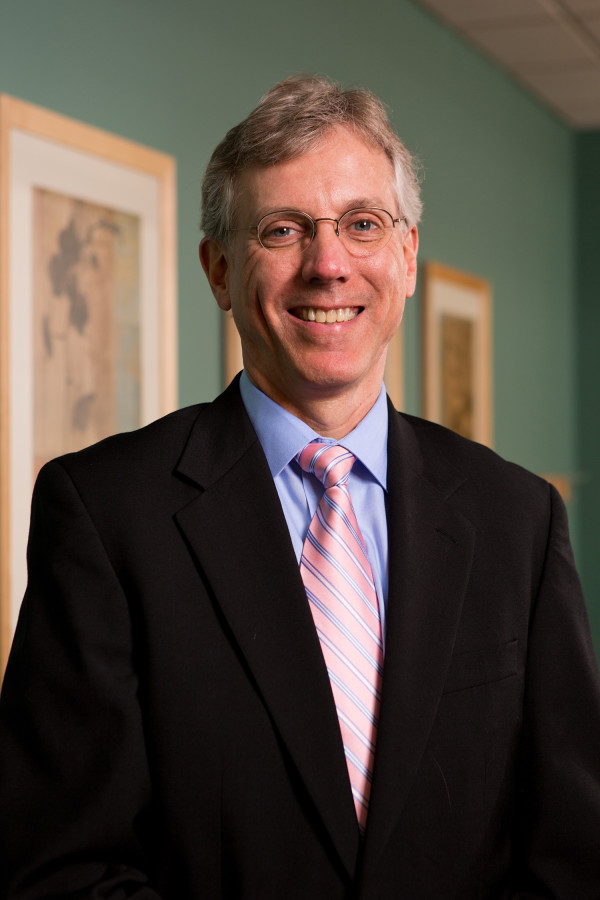
**Daniel F Hayes is the Stuart B Padnos Professor of Breast Cancer Research and co-Director of the Breast Oncology Program at the University of Michigan Comprehensive Cancer Center.** His research interests are in clinical and translational breast cancer research regarding new drug development, clinical trials and the development of biomarkers. Dr Hayes and colleagues published the first reports concerning the development of the CA15-3 blood test, used to evaluate patients with breast cancer. He has expertise in the use of this and other tumor markers, such as HER-2, circulating tumor cells and pharmacogenomics.

**Figure 2:**
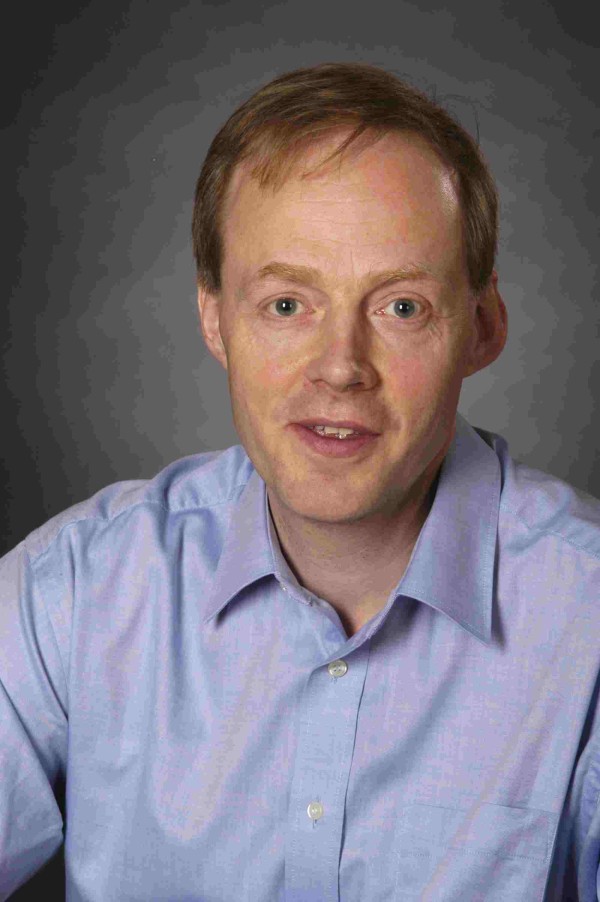
**Hugh S Markus is a Professor of Stroke Medicine and Honorary Consultant Neurologist at the University of Cambridge.** He specializes in care of patients with stroke. He runs a specialist clinic for patients with genetic causes of stroke, including cerebral autosomal-dominant arteriopathy with subcortical infarcts and leukoencephalopathy (CADASIL). His research interests are in using genetic and imaging techniques to investigate the pathogenesis of stroke.

**Figure 3:**
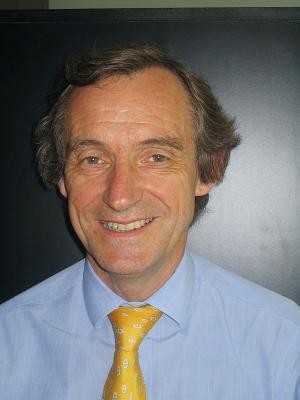
**David Leslie is consultant physician at St Bartholomew’s Hospital, Professor of Diabetes and Autoimmunity at the Blizard Institute, University of London.** His research involves studies using major patient resources (such as patients with adult-onset autoimmune disease and twins with diabetes), allied to cell and molecular studies.

**Figure 4:**
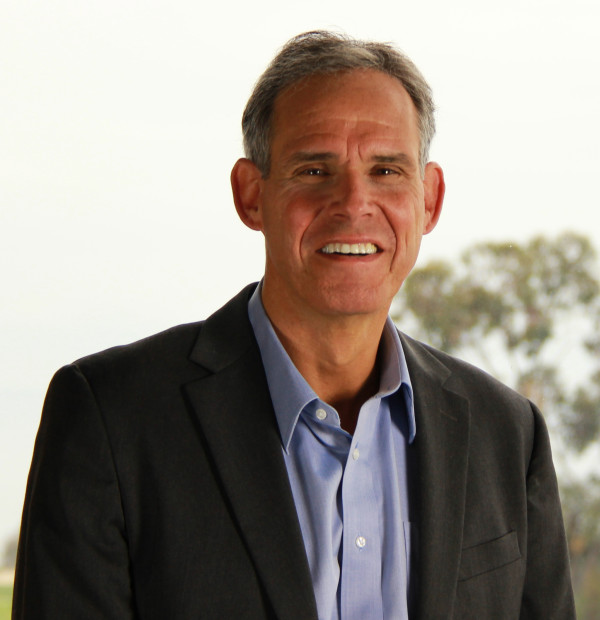
**Eric J Topol is the Director and Professor of Genomics at Scripps Translational Science Institute, co-Founder of the West Wireless Health Institute, and serves as Chief Academic Officer of Scripps Health.** He is a practicing cardiologist, having led many worldwide clinical trials to advance care for patients with heart disease, and works on genomic and wireless digital innovative technologies to shape the future of individualized medicine.

Oncology is an area that I believe has the greatest potential for the personalization of medicine, but probably has had the least application until recently. Over the last 40 or 50 years, especially in medical oncology, we have taken a ‘one size fits all’ approach; our only efforts at personalization have been trying to tailor specific chemotherapies based on the tissue of origin. For example, we treat breast cancer with slightly different drugs than we treat lung cancer. Frankly, these distinctions are not very great. The only other effort to individualize treatment is to give the chemotherapy based on the height and weight of the individual patient - so we try to get the dose approximately right. In both cases, we almost certainly overtreat many patients, give treatments that are not going to work, and under- or overdose patients. So there is a lot of room to improve treatments in medical oncology.

The reason I believe medical oncology has the greatest potential for personalized medicine is, because unlike many other diseases, we have fairly easy access to the somatic tissue that we are trying to address. For example, the cardiologist cannot carry out heart biopsies very easily. The general internists who are treating hypertension have put efforts into individualized treatment based on inherited genetic single nucleotide polymorphisms but, so far, that field has not advanced very far. In cancer, on the other hand, we have access to the tumor and we can look for various things the cancer is doing that is different from normal tissue and try to focus on that. The result of these studies over the last few years has been so-called targeted therapy.

In fact, my earlier cynical comments notwithstanding, targeted therapy has been used in oncology for the last 120 years or so, starting with Sir George Beatson, who was a Scottish physician and surgeon. He raised dairy goats and pioneered the hypothesis that there might be some connection between the ovaries and the breasts. He removed the ovaries from three women with breast cancer and reported that two of them had what we would now call a response. Beatson was removing the growth factor estrogen from tissue expressing the estrogen receptor that needed estrogen to grow [1]. It took 60 or 70 years to learn the molecular biology behind Beatson’s observation that has allowed us to, for example, focus endocrine therapy on people who are likely to benefit from it, that is, those whose cancer is estrogen receptor-positive. Endocrine therapy is not given to people who are not likely to benefit (estrogen receptor-negative).

More recently, in breast cancer, we have been able to offer more personalized treatment targeting the HER2 molecule; there are now many therapies, starting with trastuzumab, directed against HER2 [2]. I think the success with HER2-targeted therapies in breast cancer has now led to progress in a number of other diseases. For example, in chronic myelogenous leukemia, the translocation of the so-called ‘Philadelphia chromosome’ is well known, the microbiology behind it is fully understood, and we now have many drugs - starting with gefitinib - against it, which have been quite successful [3]. So I think the door of targeted personalized therapies is just starting to open and the floodgates will open in the next few years.

There are some obstacles associated with introducing so-called “personalized medicine” into oncology. The concern we have is really threefold. Firstly, we have to be certain that the individual test or biomarker for studying is accurate, reproducible and reliable. Whereas drugs are very carefully controlled, either by the US Food and Drug Administration or by various regulatory bodies, there is no similar regulatory oversight for tumor biomarker tests. Many tests that have been developed are not necessarily vetted analytically, and so it is not clear whether you can depend on them. Some tests are analytically validated independently, but many are not.

The second obstacle is that the clinical data that have been generated are often from what we call ‘studies of convenience’; somebody has some samples and someone else has an assay, and results are generated without really addressing what the question was to begin with. As a doctor and as a patient, it is not enough to know that a marker separates one population into two with statistical significance; the important question is whether the marker does it in a way that is important clinically. It is essential to know whether I would withhold therapy from one patient or give therapy to another patient because the results have shown that those patients are separate and that giving the therapy or withholding it improves their outcomes. We call that ‘clinical utility’. There are actually precious few tumor biomarker tests that have demonstrated both analytical validity and clinical utility. We are trying to encourage researchers to approach tumor biomarker tests in the same way that we approach drugs so that we can hasten introduction of these tests into the clinic because there will be a better set of ground rules.

The genomics revolution has great potential in the development of personalized oncology; I think it will revolutionize us in two ways. Firstly, there are already tests based on so-called ‘signatures of expression’, particularly with expression of RNA. For example, you can put 10,000 genes into the mix and come up with a genomic signature that can predict a patient’s outcome. This approach also has limitations; it is not yet good enough. We really need a signature that can guide treatment decisions.

Indeed, there have been very few assays that have come out of the genomics revolution in the last 12 years. One of the most highly proven and adopted is the ‘21 gene recurrence score’ for breast cancer [4]. There is really good evidence to show that if an estrogen receptor-positive node-negative patient has a low recurrence score, her odds of the cancer recurring over the next 10 years - assuming she will get endocrine therapy - are so low that even if chemotherapy works, it will not help enough people to outweigh the potentially life-threatening side effects of chemotherapy. This assay is widely used in the US and we have seen an approximately 20% reduction in the use of chemotherapy for that group of patients over the last 10 years [5]. In my opinion, this is exactly what we are trying to do with patient-tailored therapy.

The next step is next-generation sequencing, the ability to sequence the genome of an entire tumor and then compare what is found in the tumor to what is found in the patient’s normal germ line DNA. The aim is to find mutations or copy number variations, amplifications or deletions. We can look for changes for which we either already know that there are drugs that work, or place the patient on trials that have drugs that are directed towards that specific change. Next-generation sequencing is really in its infancy; it is a year or two old. Sequencing is becoming a great deal cheaper over time; as a result there are many studies being generated in which patients’ cancers are being biopsied, fully sequenced in one way or another, and then compared to their germ line DNA to see whether or not that patient might be more specifically treated. There are caveats to this approach. A major concern is how accurate these assays are analytically. Just like any test, mistakes can be made in genome sequencing. Moreover, these methods will detect genetic abnormalities that may or may not actually have biological or, more importantly, clinical significance. The second caveat is whether or not there are context-specific responses to our drugs. In other words, a mutation in, say, HER2 may be important for drug X in breast cancer, but that same mutation in lung cancer may not have the same biology because there are tissue-specific factors at play.

### Looking into the future of personalized oncology

I have three visions. The first is something we call the ‘vicious cycle of tumor biomarker generation’. The vicious cycle involves an inconsistent regulatory environment that has confused people as to how they should develop new tests, as well as insufficient reimbursement for a biomarker test [6]. I believe that the people who design and develop tumor biomarker tests should be in line for the kind of reimbursements that people developing drugs are. This does not happen at present, which creates a huge obstacle to generating high levels of evidence for these tests. In fact, the Institute of Medicine addressed some of the issues surrounding development of omics-based tests [7]. Although this report was not specific to cancer biomarker tests, it laid out a roadmap for investigators to follow, ranging from discovery to test development to demonstration of clinical utility.

Another important issue is how investigators report tumor biomarker tests. The hallmark of the scientific method is reproducibility, yet often for may published reports of putative tumor biomarker tests it is very difficult to determine the fundamental components of the research methodology. In this regard, there have been several efforts to standardize reporting criteria for pre-analytical, analytical and clinical research strategies in these reports [8].

My second vision for the future is the next-generation sequencing approach. This is the future of oncology, and we are really looking forward to that. The third area for the future of personalized oncology, I think, is not so much a scientific revolution as it is a sociologic revolution, and that is the issue of being able to look at so-called ‘big data’. With the advent of the use of electronic medical records in many medical practices around the US, we are going to have the ability to potentially review millions of patients’ outcomes in the future and apply the lessons we have learned from previous retrospective analyses of how patients do when they are treated in various ways. I hope that such approaches will remain complementary to clinical trials, so that we can generate prospective data from clinical trials but also do much better comparative effectiveness research by having access to huge databases.

### Competing interests

DFH receives support for laboratory and clinical research from Janssen Diagnostics, manufacturer of the CellSearch© system. He has three patent applications pending regarding capture and characteristics of circulating tumor cells.

## Stroke genetics: understanding disease and prospects for personalized medicine

Hugh S Markus

It is still very early days in the genetics of stroke, but recent studies from genome-wide association studies (GWAS) are telling us some quite interesting things about stroke [9]. First of all, they are telling us that different types of stroke, or different stroke mechanisms, have quite different genetic backgrounds or architecture. For example, the genes involved in large artery stroke (due to narrowing of the arteries to the brain) seem to be quite different from the genes involved in stroke associated with cardioembolism - blood clots coming from the heart. The results from GWAS are increasing our understanding of the pathophysiology of stroke. This may have important implications outside genetics. For example, it suggests that we may need to tailor our stroke treatments rather more towards different types of stroke than we do currently.

GWAS results are also beginning to tell us about new pathways that may be involved in causing stroke. Perhaps the most exciting one so far is a gene called *histone deacetylase 9 (HDAC9)*, which appears to be associated with large artery – or atherosclerotic – stroke. This may give us a new way of understanding the cause of this type of stroke, which could potentially be targeted with different treatment approaches. But this is a little way off at the moment.

The field of stroke genetics is not as advanced as other areas of medicine, for example oncology, in terms of personalized risk prediction and therapy for a number of reasons. First of all, the genes involved in stroke risk all contribute just a small amount of increased risk. Unlike some of the cancer genes, where a particular genetic mutation substantially increases the risk of developing a certain type of cancer, in stroke it appears likely that there are lots and lots of different genes that are all contributing, presumably, a small amount of risk. Therefore, if you just look at one of these variants, it is not going to give you the same sort of information as you would obtain from perhaps looking at a cancer gene. Another reason is that we have been quite late in doing GWAS genetic studies in stroke and we are only just beginning to scratch the surface. Many other diseases have been looking at genetics with the GWAS approach for rather longer, with much larger sample sizes. It may well be that we can predict stroke risk in the future but at the moment, because each of the identified genes only contributes a very small amount of risk, we are still unable to explain many of the genetic risk factors. Therefore, looking at just a few genes, when there are many we do not understand or have not yet discovered, is only going to contribute a limited amount to overall risk.

### The outlook for stroke prediction

In the future, genetics could be used to tailor treatment for the individual patient in a number of possible ways. One approach is in looking at pharmacogenomics. This is where people respond differently to treatments according to their genetic makeup. Tailored treatment is already applied with drugs such as the anti-platelet agent clopidogrel, which is frequently used to reduce recurrent stroke, and warfarin, an anticoagulant that is often used to prevent recurrent stroke in patients with, for example, atrial fibrillation. For both of these treatments, there are genetic variants that determine how the body breaks down the drug and therefore what sort of dose of the drug you are going to need or, in the case of clopidogrel, whether you are likely to respond well to the drug or not. Therefore, there is already exciting data that suggest you can look at these genetic variants to tailor doses to individual people.

There has been considerable debate about how useful these genetic tests are, particularly in the US where there were recommendations, for example, that one should test for clopidogrel sensitivity before giving clopidogrel. What we really need to know is whether adopting a program where you test individual patients’ genes and use it to guide therapy actually results in improved outcome for patients. This still has to be proven. In the UK, pharmacogenomics tools are not being used very widely at the moment. Whether pharmacogenomics actually has a major impact on outcome and allows us to reduce recurrent stroke risk is currently being determined in clinical trials. If such trials show they really do predict outcome then pharmacogenomics may be used more widely.

Another important question is whether we can predict overall stroke risk in individual people. Because we only know a few of the genes that are contributing to overall genetic risk, this is a little way off. When many more genetic studies with larger sample sizes have been carried out and we can explain more of the overall genetic risk, it may well be that we can start predicting risk in individual people. The question then is whether we can predict usefully more risk than we can with conventional cardiovascular risk factors. For example, we know that high blood pressure, smoking and having high cholesterol are linked to an increased risk of stroke. The question is, can genetics predict more risk than these factors, and how useful is it? We will only be able to answer this when we have discovered more of the overall genetic component for stroke.

### Competing interests

HM has no competing interests to declare.

## Diabetes management: targeting the right treatment to the right patient

R David Leslie

Diabetes management is becoming more tailored towards the individual patient in terms of the treatment that is available and what that treatment does for the patient. Twenty years ago, there were a very limited number of drugs available for patient care. That is, we had insulin, sulfonylureas and diguanylates. Our approach to the management of patient care was entirely focused around the prevention of complications. We tended to give every patient with diabetes a standard form of therapy, which was usually with one or other of those drugs, and we always gave those drugs in the context of trying to prevent progression of complications by reducing blood glucose. Diabetes management employed a very glucose-centric approach in the past.

The number of drugs available to us has now increased substantially and, as a result, the approaches to therapy have become more diverse; we now have more options in terms of drug therapy. Also, drug therapy available previously was rather non-specific; everyone was given the same treatment. However, we are now beginning to identify sub-groups of people with diabetes in which individuals are more responsive to some of these agents, or for which these agents are more suitable. We still, unfortunately, are constrained by our approach, which is the prevention of complications, but we have expanded our horizons so that while before we had a glucose-centric approach, now we focus not only on glucose but also on smoking, hypertension, high blood cholesterol and exercise [10].

The guidelines for managing diabetes have evolved in recent years, and there are arguments for adopting patient-tailored rather than guideline-led treatment strategies [11]. A guideline approach is very convenient for someone who is not an expert because it gives, quite literally, a guideline as to which direction you might go and what drugs you might use. Professionals who are experts in that field should not require a guideline. They would know the condition and the person in front of them - that is the very nature of a professional interaction. The problem is that many guidelines are taken as tablets or commandments; with the overwhelming number of cases of diabetes, non-experts deal with cases in which guidelines are probably quite valuable to them because it gives them a broad-brush approach to the condition, without being able to modify or refine their management policy [12]. Another problem is that there is a substantial lag between where we are as specialists and where the guidelines are, so inevitably there will be positions taken by specialists that the people who produce guidelines are not going to have had time to illustrate. We have this problem with books. I have just published a text book, but I am sort of wondering whether I should be producing any more because by the time you get round to publishing - inevitably it has a two-year gestation, rather like a guideline - things have changed. You started with one form of therapy and you have moved on to another.

Currently, most diabetes drugs address insulin secretion; very few address insulin sensitivity. This is a broad area where we might be able to target people who have a particularly severe problem with insulin sensitivity, where their requirement for drugs is distinct from those whose predominant problem is its secretion. We already know that patients with adult-onset autoimmune diabetes who do not require insulin initially seem to do badly with sulfonylureas, yet sulfonylureas would be considered to be the second line of treatment. Certainly all the guidelines say as much. By way of contrast, there is another type of diabetes associated with a genetic mutation, maturity-onset diabetes of the young, in which sulfonylurea is particularly prone to causing hypoglycemia, and it is therefore necessary to give these patients a slightly different agent. However, there is a really rare condition where the potassium channel of the beta cell has a mutation that can be circumvented by sulfonylureas [11]. These are all examples of how our understanding of the pathophysiology and the genetics of the disease can be applied to target the appropriate treatment. Now, there is an additional issue with side effects: the more drugs you use, the more likely you are to get side effects [10,13,14]. A lot of our patients get side effects. Of course, the more drugs you have available, the greater the number of options. So if you have a particular side effect in a particular individual - or a risk of a side effect - then it may be that you will look at a different drug for that individual [12,15].

In addition to there being more drugs available, there have also been changes in the targets that we have. For example, someone who is 80 will have completely different targets for blood glucose compared with someone who is 20. This is because the person who is 80 is at high risk of macrovascular complications, whereas the person who is 20 is at risk of both macrovascular and microvascular complications [11,15,16]. This is another example of personalized medicine. Because we have different therapies and different targets for therapies and different approaches to therapies, so we will start seeing a more focused approach in the physicians who are understanding and treating the disease.

### Prospects for diabetes management

Looking forwards, I think the future of this field is very substantial - and when I say this field, I mean the concept of personalized medicine in general as compared with our current guidelines. I think this extends far beyond diabetes into a wide range of medical conditions. The two key questions are: what are we going to use and why are we going to use it? In terms of what we are going to use, as we understand more about the heterogeneity of the disease, then we will be able to target the increasing number of drugs that are available to a particular individual with a particular condition [10,17,18]. As we find more about the genetics that underline the process of diabetes, and as we identify more how one person with diabetes differs from another, so we will be able to identify drugs that are of specific value in that individual.

### Competing interests

DL is currently on advisory boards for Novo Nordisk, Diamyd and Andromeda.

## Innovations and legislations in mobile health apps: are they suitable for all?

Eric J Topol

The field of mobile health has advanced very rapidly in the last few years. If you look back at the first 10 years of this century, these little devices - starting with iPods and quickly migrating through smart phones, e-readers and tablets - have changed our world. That was in terms of day-to-day living, but now we are starting to see the same thing in medicine; these little mobile devices having a profound and really a transformative effect on the future of healthcare [19].

The smartphone will become the hub of future medicine, because it has a pluripotent impact. For one, it can be the conduit of sensor information. Smartphones can be used to measure blood pressure, glucose, heart rhythm and brain waves; the list is almost endless. In addition to biosensors, we also have the ability to change a mobile phone into a scanner - an otoscope, ophthalmoscope, microscope or any kind of scope - and in fact there are even little ultrasound devices that can function as a mobile phone. These are all examples of how mobile phones can be used as medical sensors. Another way the smartphone can be used in medicine is by functioning as a laboratory on a chip, which is capable of doing almost any common laboratory assay. These include tests for kidney function, liver function, thyroid function, blood thinning international normalized ratio, potassium, and many more. These are just a few ways that smartphones can be used for collecting data and capturing information for a particular individual, to shape that person’s care.

An important consideration in the advancing field of mobile health is safety and regulation of smartphone apps. The US Food and Drugs Administration recently released a statement on this issue to say that most smartphone apps do not require their oversight. The only ones requiring regulation are those that make critical measurements such as blood glucose, blood pressure and heart rhythm - these are important devices. For example, an error in glucose measurement could result in a patient taking a lot of insulin, leading to coma, seizures or even death. Devices that make critical measurements are not just apps, they are also add-ons to the phone; they involve hardware and there has to be some demonstration that accuracy has been fulfilled. I think it is vital that we have an independent agency, a regulatory body that can assure that the things being measured are being done so in a highly rigorous, accurate way.

After mobile medical devices have been approved, we must determine whether they are suitable for people without medical training to use. Smartphones are immensely popular; there are more cell phones in the world - by a great margin - than the number of people, toilets and toothbrushes. In the present day, a large proportion of people who own a cell phone have a smartphone. Therefore, we would like apps that are used for medical tracking to be very consumer-oriented with a friendly user interface. Of course some of them have that, such as those that measure blood pressure and glucose. Some mobile medical devices are really showing that making critical measurements can be achieved without any medical training, and that is of course the path that mobile health needs to go on.

There are a few potential risks associated with using mobile apps in healthcare that are important to mention. Firstly, lack of accuracy is a key concern. For example, if a mobile device was accurate and then started to lose its calibration, this would be an important safety issue. Many apps do not require subsequent calibration after coming out of the factory. However, some do require monitoring, and in these cases there would be specific recommendations about whether there is any potential for drift. For example, if a device functions as a glucose sensor, it is necessary to re-check the calibration with a conventional finger stick at x number of days to ensure that there has been no drift.

A second concern would be security of the data; potential problems could be escapes or re-identification of data that are supposed to be kept de-identified. This issue has to be addressed because anything that is digitized can be hacked into or breached, so data privacy is a concern. The third level of risk is inducing severe anxiety. That is, it is very difficult for some people to have their own data continuously displayed on their device. People who would worry about their data would tend to be highly anxious anyway, and this continuous monitoring would create another cause of anxiety at a high level. It is important to establish which patients are not suitable for this, and how we can reduce this type of anxiety. As a doctor, it is important to realize that mobile health is not good for all people. You have to figure out which individuals will benefit from continuous monitoring of their health.

The big question about using smartphones in medicine is whether they can change behavior and encourage people to live healthier lifestyles; that is the hardest thing to do. However, in the era of mobile health, we have real-time, continuous data, passively being captured. What remains to be seen is whether or not that will indeed push people to become more healthy in their lifestyle. There is a great deal of hope for this approach, and there are ways that people can be incentivized to be more healthy; this can be done through smartphone games and managed competition. There are all sorts of ways in which having one’s own data could be a stimulus to living a better lifestyle and, ideally, preventing the development of a chronic condition.

### The promise of mobile health

I think this is the most exciting time in the history of medicine and it is because of mobile health. I think that, just as medicine is moving into crisis mode in economic terms, fortunately at the same time the area of mobile health is blossoming, exploding with innovation. It has a lot of promise for lowering costs for the first time, which has never really been the case with new technology in medicine. Because mobile health is consumer-based - it is a consumer cell phone, and people have access to their own data - it is particularly enthralling for me to see this shift away from the doctor-dominated world of medicine to a much better parity and symmetry of information; the flow of information directly to the patient and then the guidance from the physician. I am really impressed with how far this field has come already in such a short time and where it can go. In the era of mobile medical apps, the goal is ultimately to reduce the number of times patients have to visit the doctor. So much of this information can be acquired anywhere at any time, so in the future it may not be necessary to go to the doctor to get a blood pressure check or a heart rhythm check. Many measurements could be carried out by oneself, at people’s own convenience, and much more data could be collected than ever before.

The hope is that mobile health will lead to a marked reduction in resource consumption and emergency room use, and ultimately there will be fewer people in the hospital because they can have their vital signs and critical measurements assessed anywhere. We hope that the only people in hospital will be those who are acutely ill in the intensive care unit, or those who are there for a special procedure or operation. But mostly, the majority of people will be at their home, which is much safer from the standpoint of infection and medication errors, and much cheaper. What is more, patients will be able to sleep in their own bed, while previously it was necessary to stay in hospital. I envision mobile health will give rise to the end of the hospital as we know it today, and the end of office visits as we know them today; these will not end entirely, but will have a radical re-booting.

### Competing interests

ET is on the Board of Directors for Dexcom, is an advisor for Sotera Wireless, and is on the Scientific Advisory Board for Quanttus.

A podcast on the topic is available at http://www.biomedcentral.com/biome/personalized-medicine-risk-prediction-targeted-therapies-and-mobile-health-technology
